# Advancing respiratory virus diagnostics: integrating the nasal IFN-I score for improved viral detection

**DOI:** 10.1016/j.ebiom.2024.105450

**Published:** 2024-11-21

**Authors:** Marine Mommert-Tripon, Delphine Parraud, Cloé Grosbois, Alexandre Gaymard, Valérie Cheynet, Bruno Lina, Guy Oriol, Frédéric Laurent, Caroline Dupré, Quentin Semanas, Antonin Bal, Laurence Generenaz, Sylvie Pons, Karen Brengel-Pesce, Audrey Guichard, William Mouton, Florence Morfin, Aurore Fleurie, Sophie Trouillet-Assant

**Affiliations:** a Joint Research Unit Civils Hospices of Lyon-bioMérieux, Hospices Civils de Lyon, Lyon Sud Hospital, Pierre-Bénite, 69310, France; b Open Innovation & Partnerships (OIP), BioMérieux S.A., Marcy l’Etoile, 69280, France; c Infective Agents Institute, Hospices Civils de Lyon, Croix-Rousse Hospital, Lyon, 69004, France; d International Center of Research in Infectiology, Virpath Team, Lyon University, INSERM U1111, CNRS UMR 5308, ENS, UCBL, Lyon, 69000, France

**Keywords:** Interferon, Respiratory viral infections, Diagnostic, Biomarker, Host response

## Abstract

**Background:**

This study aimed to demonstrate the utility of the nasal Type I interferon (IFN-I) response as a marker for respiratory viral infections (RVIs) and its potential to enhance diagnosis when combined with first-line PCR tests for Influenza A/B, RSV, and SARS-CoV-2.

**Methods:**

Nasopharyngeal swabs (NPS) from patients at Hospices Civils de Lyon (November 2022–April 2024) suspected of viral infections (n = 788) and from healthy controls (n = 53) were analysed. The IFN-I score was measured using the FILMARRAY® IFN-I pouch prototype, which detects four interferon-stimulated genes. The study evaluated the performance of the IFN-I score in detecting samples positive for viruses by first-line PCR and assessed its benefit in diagnosing RVIs in samples initially classified as negative by PCR.

**Findings:**

Out of 788 NPS included, 504 (64%) were positive with the first-line PCR tests, and IFN-I score was significantly higher in those samples (median [IQR]: 13.00 [2.76–45.40]) compared to ones collected from healthy controls (1.09 [0.67–1.30]; p < 0.0001), with an area under the curve (AUC; 95% CI) of 0.92 (0.90–0.92). Moreover, out of the 284 NPS negative with first-line PCR tests, suspicion of viral infection according to IFN-I score was found in 63% of cases (178/284). Second-line test (BioFire**®** Respiratory Panel 2.1 *plus*) and viral metagenomic confirmed the presence of viruses 94% of cases.

**Interpretation:**

The study highlights the potential of integrating nasal IFN-I score into clinical workflows to improve RVI diagnosis and enhance preparedness for emerging viruses.

**Funding:**

Public grant overseen by the 10.13039/501100001665French National Research Agency (ANR21-RHUS-08/ANR-23-CHIN-0001).

**Research in context**

**Evidence before this study**

We searched PubMed/MEDLINE on October 14th 2024, using the search terms “host response”, “transcriptomic signature”, “diagnostic”, and “respiratory viral infections” with the “Humans” filter applied. Of 85 publications, 77 studies were either not directly focused on diagnostic and/or limited to a specific virus infection (SARS-CoV-2, influenza, RSV, tuberculosis). Among the 8 remaining publications, various host response-based signatures were discovered. However, none of these studies demonstrated the performance of an easy-to-use diagnostic tool for viral infections using nasopharyngeal swabs on a comprehensive range of respiratory viruses in both adult and paediatric populations.

**Added value of this study**

Host response-based biomarkers are very promising, and numerous studies have highlighted their value in diagnosing respiratory viral infections using whole blood samples, such as FebriDx and MEMED. However scientific and medical communities advocate for the use of non-invasive methods such as nasopharyngeal swabs. This study demonstrated the diagnostic value of the Type I interferon response for respiratory viral infections using a nasopharyngeal swab-based test, which could be seamlessly integrated as a complementary tool to first-line tests in routine virology laboratories.

**Implications of all the available evidence**

The present study underlines the importance of a diagnostic tool based on host response rather than pathogens screening only for respiratory viral infections diagnosis. The monitoring of nasal Type I interferon response represents a marker of active respiratory viral infections offering new perspectives to improve management of patients with respiratory infections and to enhance preparedness for emerging viruses.

## Introduction

Respiratory tract infections pose significant public health challenges and impose substantial morbidity and mortality burdens on both children and adults worldwide. The primary pathogens involved are viruses, the most frequent of which are influenza viruses [influenza A (IAV), influenza B (IBV)], respiratory syncytial virus (RSV), rhinovirus (HRV), and coronaviruses.^1^ Highly transmissible and contagious, such infections can be the cause of seasonal epidemics or pandemics.^2^ Current diagnostic strategies mainly use PCR-based identification targeting each type of virus. However, because of the huge diversity of respiratory pathogens, routine laboratory PCR-based tests for the systematic detection of all pathogens likely to be involved in respiratory infections using such specific PCRs appears tedious and expensive despite multiplexing options.^3^ For this reason, several international and national recommendations suggest limiting the investigation of the aetiology of the most frequent acute respiratory infections using molecular biology tests, either multiplex or combined, and including severe acute respiratory syndrome coronavirus 2 (SARS-CoV-2), influenza viruses, and/or RSV.^4^, ^5^, ^6^ These represent the first-line of tests performed by routine virology laboratories particularly during epidemic periods. However, less commonly tested respiratory viruses can also cause acute lower respiratory tract infections (LRTIs) in both children and adults, potentially leading to hospitalisation or death. These include human metapneumovirus (hMPV), adenovirus (ADV), and HRV.^7^, ^8^, ^9^ It is therefore crucial to develop alternative, reliable, and rapid diagnostic tools that can better guide respiratory viral infection (RVI) diagnosis and enhance the comprehensive determination of RVI aetiology and patient management.

In the fields of bacteriology and mycology, universal PCR is possible because the 16S and 18S genes, respectively, are shared.^10^ In virology, pathogens do not share genomic structure; their only common characteristic is the capacity to induce a type I interferon (IFN-I) response. Upon detection of viral particles by innate immunity receptors, an antiviral response is initiated, leading to the transcriptional induction of type I and III interferons (IFNs). These IFNs are secreted and recognised by their respective cellular receptors (IFNAR1/2 for IFN-I, and IFNLR/IL10RB for IFN-III), activating a JAK/STAT-mediated signalling pathway.^11^ This activation results in the expression of IFN-stimulated genes (ISGs), which encode more than 700 proteins with antiviral properties.^12^^,^^13^ However, since IFN-I proteins are secreted at very low concentrations (i.e., in the femtomolar range), they can only be detected using ultrasensitive assays, which are not widely deployed in routine laboratories.^14^^,^^15^ In this context, alternative methods have been proposed,^16^^,^^17^ such as detecting the IFN-γ-inducible protein 10 (IP-10) cytokine as a proxy of the IFN-I response.^18^ Quantifying this protein in nasopharyngeal swabs (NPS) has been identified as a promising marker for RVIs caused by human rhinovirus, seasonal Coronavirus (CoV-NL63), and SARS-CoV-2.^18^ Furthermore, the transcriptomic approach could provide an alternative method to assess the IFN-I response. For example, Ng et al. demonstrated that patients with various RVIs (SARS-CoV-2, IAV, RSV, hCoV) exhibited an up-regulation of the IFN-I pathway compared to controls without infection, using an RNA-seq approach in the nasal mucosa.^19^ In addition, we have previously reported the value of a nasal IFN-I score based on measuring a limited number of ISGs transcripts within NPS for diagnosing SARS-CoV-2 infection.^20^^,^^21^

The aim of the present study was to assess whether the nasal IFN-I response could serve as a meaningful marker for RVIs and enhance RVI diagnosis when combined with first-line PCR tests.

## Methods

### Study design

**Fig. 1:**
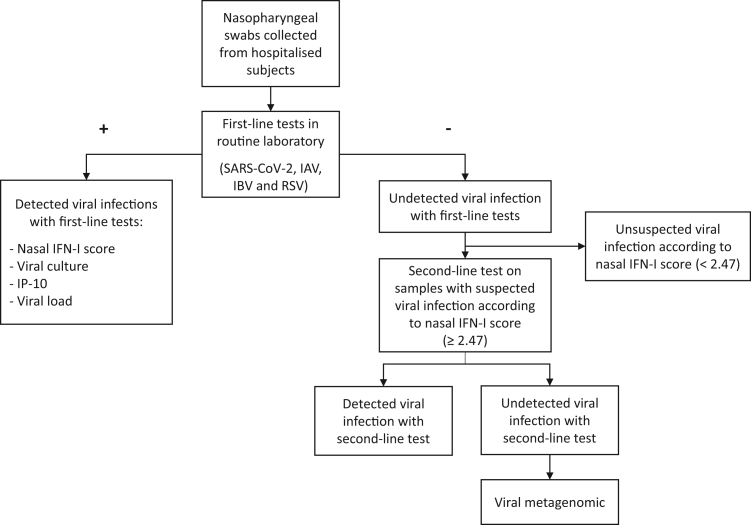
**Workflow of the study**. Initial virus screening involved first-line assays for detecting SARS-CoV-2, IAV, IBV, and RSV, following standard procedures. For samples testing positive (left side), the nasal IFN-I score, viral culture, IP-10 quantification, and viral load were assessed to evaluate the nasal IFN-I score's effectiveness. Samples testing negative (right side) underwent further evaluation using the nasal IFN-I score, with a score of ≥2.47 indicating a possible viral infection, prompting the second-line tests (BioFire**®** Respiratory Panel 2.1 *plus***)** and viral metagenomics analysis. IAV, influenza A virus. IBV, influenza B virus. IFN-I, type I interferon. IP-10, IFN-y-inducible protein 10. RSV, respiratory syncytial virus. SARS-CoV-2, severe acute respiratory syndrome coronavirus 2.

NPS (UTM; Copan Diagnostics Inc., Carlsbad, CA, USA) collected from patients suspected of RVIs during their hospital stay at the Hospices Civils de Lyon and sent to routine virological laboratory for virological diagnosis were included in the RESPIFERON study, reported herein, between November 2022 and April 2024. Initial virus screening was performed using first-line assays for the detection of SARS-CoV-2, IAV, IBV, and RSV according to routine laboratory procedures and national recommendations. On NPS samples that tested positive for at least one virus detected by the first-line tests, we determined the nasal IFN-I score, conducted viral culture, as well as quantified IP-10 and viral load to evaluate the performance of the nasal IFN-I score (Fig. 1). For NPS samples that yielded negative results in the first-line tests, the nasal IFN-I score was employed to assess the likelihood of a viral infection. When the nasal IFN-I score was elevated (≥2.47) a viral infection was suspected, and these samples underwent second-line tests and viral metagenomics analysis (Fig. 1).

In addition, NPS collected from asymptomatic subjects for SARS-CoV-2 screening before hospital admission were used as healthy controls (HCs). Noteworthy, these samples were negative for all the viruses tested using first- and second-line tests.

Of note, 500 μL of NPS samples were diluted in 500 μL of lysis buffer (NucliSENS® easyMAG®; bioMérieux, Lyon, France) to stabilise nucleic acids before storage at −80 °C.

### Ethics et regulatory measures

The data processing associated with this study complied with the General Data Protection Regulation (GDPR) and is registered in the data controller's data processing register (HCL) under the number 21_5647, it was also approved by the scientific and ethics committee of the Hospices Civils de Lyon (IRB n°00013204) under the number N°21_647. The study is registered under clinical trial number NTC06017310. In agreement with the GDPR [Regulation (EU) 2016/679 and Directive 95/46/EC] and French data protection law (Law n°78-17 on 06/01/1978 and Decret n °2019-536 on 29/05/2019), a non-opposition consent form was sent to all patients. All samples were fully anonymised before analysis.

### Type I interferon response

The IFN-I response was assessed using a FILMARRAY® IFN-I pouch prototype provided specifically by bioMérieux for this study; this allows the measurement of four ISGs [interferon alpha inducible protein 27 (*IFI27*), interferon-induced protein 44 like (*IFI44L*), interferon-induced protein with tetratricopeptide repeats 1 (*IFIT1*), and radical S-adenosyl methionine domain containing 2 (*RSAD2*)] and three housekeeping genes [hypoxanthine phosphoribosyltransferase 1 (*HPRT1*), peptidylprolyl isomerase B (*PPIB*), and 2,4-dienoyl-CoA reductase 1 (*DECR1*)] for signal normalisation (this prototype has not been submitted to any regulatory agency for review at the time of writing). A total of 100 μL of diluted NPS was tested using the prototype IFN-I pouch as described previously.^21^ The normalised expression value of each assay was then computed using the housekeeping genes. The nasal IFN-I score was calculated as previously described.^22^ Briefly, the relative expression was determined for each normalised ISG expression dividing by the median normalised expression of each ISG from a control group. Finally, the median of these four ISGs relative expression was used to calculate the nasal IFN-I score.

### Cytokine quantification

The concentrations of IP-10 were measured in diluted NPS using the Simple Plex human CXCL10/IP-10 assay kit in the ELLA Automated Immunoassay System (ProteinSimple, San Jose, CA, USA), according to the manufacturer's instructions. All values below the lower limit of quantification (LLOQ = 0.94 pg/mL) were reduced to a value equal to LLOQ/√2 (=0.349 pg/mL) to perform statistical analyses.

### Virological investigations

#### First-line virus screening

NPS were tested in the routine laboratory using the real-time RT-PCR SARS-CoV-2/IAV/IBV/RSV Assay on a Panther Fusion™ (Hologic®, San Diego, CA, USA) according to the manufacturer's instruction.

#### Second-line virus screening

The detection of respiratory pathogens was performed on diluted NPS (off-label sample type for the BioFire**®** Respiratory Panel 2.1 *plus*) using the BioFire**®** Respiratory Panel 2.1 *plus* detecting 23 respiratory pathogens (bioMérieux) according to the manufacturer's instructions.

#### Viral load assessment

The nasal viral load was determined using nucleic acids previously extracted from 200 μL of diluted NPS using the NucliSENS® easyMAG® (bioMérieux). Parvovirus B19 and Cytomegalovirus amplification were performed using virus-specific R-GENE® kits (bioMérieux) and the Bio-Rad CFX96™ thermocycler (Bio-Rad, Hercules, CA, USA), according to the manufacturing instructions. The R-GENE® kits were associated with quantification standards (QS) internally developed for research use only. Viral load was then expressed as [log_10_cp/10^6^ cells].

#### Viral culture

A total of 500 μL of RT-qPCR/qPCR-positive NPS were inoculated a maximum 24 h after sampling without freezing on suitable cell lines using a culture medium appropriate for growth, i.e., on confluent VeroE6/TMPRSS2 (RRID:CVCL_YQ48), MRC-5 (RD-Biotech, France, RRID:CVCL_0440), MDCK (ATCC Cat# CCL-34, RRID:CVCL_0422), and HEP-2 cells (ATCC Cat# CCL-23, RRID:CVCL_1906). After 3, 8 and 15 passages, all cell lines are tested for mycoplasma using the MycoAlert® Mycoplasma Detection Kit (Lonza, France). Plates were incubated at 33 °C in 5% CO_2_. Cytopathic effects were then monitored daily; positive samples were harvested for the confirmation technique, while negative samples were cultured for 8 days. RNA or DNA from infected cells were harvested and were assayed using Panther Fusion® RT-PCR/PCR kits (Hologic Inc.) for viral identification, except for RSV, IAV and IBV which were detected by immunofluorescence using IMAGEN™ RSV detection kits (K610211-2, Thermo Fischer Scientific, Waltham, MA, USA), and PathoDx™ Respiratory Virus Panel Influenza A and B Reagent (R62405 and R62406, Thermo Fischer Scientific), respectively.

### Metagenomic workflow

The investigation of viruses from NPS using metagenomics was performed as previously described by Bal et al.^23^ Briefly, a no-template control (NTC) consisting of RNase-free water and MS2 bacteriophage was used to spike NPS and used as negative and positive controls, respectively. For sample viral enrichment, a 3-step method was applied to 220 μL of NPS (low-speed centrifugation, followed by the filtration of the supernatant and then Turbo DNase treatment) to check the validity of the process and to increase sensitivity of DNA and RNA virus detection and overcome human contamination. After viral enrichment, total nucleic acids were extracted using the NucliSENS EasyMAG platform (bioMérieux) and were randomly amplified using the WTA2 kit (Sigma–Aldrich, Darmstadt, Germany). After library preparation by COVIDSEQ (Illumina Inc., San Diego, CA, USA), sequencing was finally performed on a 2 × 100pb cartridge on a NovaSeq 6000 (Illumina Inc.) with XP workflow following the manufacturer's recommendations.

As previously described (preprint article),^24^ trimming and filtering of raw paired reads were performed using cutadapt (minimum length of 30 bases, min quality of q20 and error rate set at 0) followed by a dehosting step (i.e., human reads filtering out) using sra-human-scrubber. Duplicated reads were then removed by using bbmap. Taxonomic annotation of vmNGS was performed by Kraken 2 with a threshold confidence set to 0.51 to remove false positive assignments. A custom database was used; this included viral taxonomy from RVDBv24.1^25^ and Inphared databases (v2 Nov 2022, phages with an unidentified host excluded),^24^ as well as archaeal, bacterial, fungal, and human taxonomy from RefSeq databases (downloaded with kraken 2 21/09/2021). Read binning was performed by an in-house python script. The metagenomic results were submitted to the SRA database with the following accession number PRJNA1171628.

### Statistical analysis

Nasal IFN-I scores were compared using non-parametric tests, either the Wilcoxon-Mann-Whitney test (2 groups) or the Kruskal–Wallis test (>2 groups) followed by a Dunn's test for paired wise multiple comparison. For descriptive analysis, data were presented as median and interquartile range [IQR]. The Gaussian distribution followed by the nasal IFN-I scores obtained from the HCs population allowed to determine the positive threshold of the nasal IFN-I score based on the 99.9 percentile (value = 2.47) of this distribution. Diagnostic performance of the nasal IFN-I score compared to virus detection by first-line PCR tests, viral culture, and nasal IP-10 measurement were evaluated using receiver operating characteristic (ROC) and area under curve [AUC (95% confidence interval, 95% CI)]. Sensitivity and specificity, negative predictive value (NPV) and positive predictive value (PPV) were determined with the positive threshold of 2.47. The confidence intervals for sensitivity, specificity, NPV and PPV were calculated using the method described by Agresti et al.^26^ The AUC and its confidence interval, along with the estimated AUC differences and their confidence intervals for paired comparisons, were calculated using DeLong's method.^27^ All statistical analyses were conducted using R software (version 4.2.2; the R Foundation for Statistical Computing, Vienna, Austria). Adjusted p values were calculated using the Benjamini-Hochberg correction method. p values as well as adjusted p values were two-tailed and were considered as statistically significant if <0.05.

### Role of funders

This study was funding by public grants and bioMérieux, kits have been kindly provided by bioMérieux and some authors are bioMérieux employees. Those authors play a role in data collection, analysis, interpretation, and the writing of the manuscript. Academics authors declare that they have not received any compensation for conducting this research or direct any salary. Authors were not precluded from accessing data in the study, and all accept responsibility to submit for publication.

## Results

### Sensitivity of the nasal IFN-I score to detect RVIs

Among the 788 NPS included in the RESPIFERON study, 64% of samples were positive (n = 504), each containing at least one respiratory virus from the first-line panel (SARS-CoV-2, IAV, IBV and RSV). The prevalence of SARS-CoV-2 was 43%, (219/504), that of IAV 28% (143/504), RSV 25% (126/504), and IBV 8% (39/504). Of note, there were 23/504 samples (4%) with a co-infection, i.e., containing at least 2 of the 4 viruses screened with the first-line tests. In addition, 53 samples collected from asymptomatic subjects served as HCs.

**Fig. 2:**
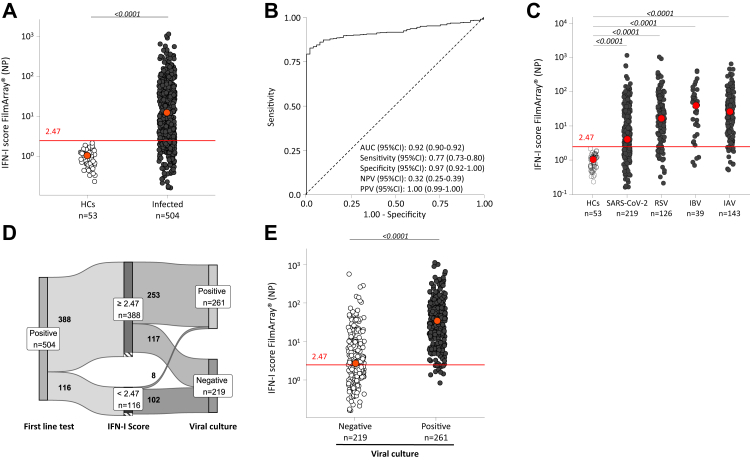
**Performance of the nasal IFN-I score to detect viral infections**. (A) Scatterplot showing nasal IFN-I score in HCs (n = 53) and patients with infection with at least one respiratory virus detected (n = 504). The red line shows the determined threshold of 2.47. The red dots correspond to median. Adjusted p values were calculated using the Benjamini-Hochberg correction method and were considered statistically significant if <0.05. (B) ROC discriminating between HCs and patients with infection. AUC, sensitivity, specificity, NPV and PPV are presented and represent the capacity of the nasal IFN-I score to discriminate HCs from patients with infection. Sensitivity, specificity, NPV and PPV were determined with the positive threshold of 2.47. (C) Scatterplot of nasal IFN-I score in the HCs group (n = 53), and in the group with infection according to respiratory virus identified with first-line test (SARS-CoV-2 n = 219, IAV n = 143, IBV n = 39, and RSV n = 126). Samples with co-infections (n = 23) are included in two different virus groups. The red line shows the determined threshold of 2.47. The red dots correspond to median. Adjusted p values were calculated using the Benjamini-Hochberg correction method and were considered statistically significant if <0.05. (D) Sankey plot showing the number of positive samples after the first-line test (n = 504) with a nasal IFN-I score above (n = 388) or below (n = 116) the threshold of 2.47. Nasal viral cultures were carried out, with 261 samples classified as positive and 219 classified as negative. Of note, nasal viral culture could not be done on 24 samples, which are represented by hatched areas. (E) Scatterplots of the nasal IFN-I score according to replicative capacity of the virus determined by nasal viral culture (negative n = 219, positive n = 261). The red line shows the determined threshold of 2.47. Adjusted p values were calculated using the Benjamini-Hochberg correction method and were considered statistically significant if <0.05. AUC, area under the curve. HCs, healthy controls. IAV, influenza A virus. IBV, influenza B virus. IFN-I, type I interferon. NPV, negative predictive value. PPV, positive predictive value. ROC, receiver operating characteristic. RSV, respiratory syncytial virus. SARS-CoV-2, severe acute respiratory syndrome coronavirus 2.

The nasal IFN-I score was calculated for each sample to assess its sensitivity to detect RVI. There was a significantly higher nasal IFN-I score in NPS of patients with infection (median [IQR], 13.00 [2.76–45.40]) compared to those of HCs (median [IQR], 1.09 [0.67–1.30]; p < 0.0001, Benjamini-Hochberg correction method), regardless of the virus detected (Fig. 2A). The AUC (95% CI) was 0.92 (0.90–0.92), indicating strong performance of the nasal IFN-I score to diagnose RVI irrespective of the virus involved (Fig. 2B). In addition, we observed variations in the score according to the virus and thus variable diagnostic performance, e.g., the median [IQR] nasal IFN-I score quantified in SARS-CoV-2 positive NPS samples (4.26 [1.74–24.60]) was six times lower than those positive for IAV (27.50 [7.08–61.40], p < 0.05, Benjamini-Hochberg correction method; Fig. 2C and Supplementary Table S1). Nevertheless, the nasal IFN-I score demonstrated a capacity to discriminate HCs from patients with infection independently of the virus involved, with AUCs (95% CI) ranging from 0.87 (0.83–0.92) for SARS-CoV-2 to 0.97 (0.95–1.00) for IAV (Supplementary Table S1).

Additionally, we noticed that 23% of samples positive for virus (116/504) had a nasal IFN-I score below the positive cut-off score of 2.47 (Fig. 2D). Nevertheless, PCR tests specific for virus could also amplify nucleic acid traces, which may lead to misinterpretation regarding the diagnosis of RVI. We then hypothesised that the low nasal IFN-I score observed in those proportion of NPS with PCR-positive could be associated with low viral load and subsequently with the replicative capacity of viruses. We observed that samples with a nasal IFN-I score greater than or equal to 2.47 had a median nasal viral load at least two times higher than samples with a nasal IFN-I score below 2.47 (p < 0.05, Wilcoxon-Mann-Whitney test; Supplementary Figure S1A). Therefore, based on viral culture results, we investigated whether the nasal IFN-I score could distinguish between replicative viral infections among samples with PCR-positive (Supplementary Figure S1B). Viral culture data were obtained for 480/504 NPS. As expected, for each virus, viral load was higher in positive viral culture (Supplementary Figure S1B). Furthermore, 88% (102/116) of the positive samples using the first-line tests but with a nasal IFN-I score below the 2.47 threshold were negative in viral culture (Fig. 2D, Supplementary Figure S1A, C). Regardless of the virus involved (SARS-CoV-2, IAV, IBV, or RSV), the median [IQR] nasal IFN-I score was thirteen-fold higher in samples with culture-positive (n = 261; 36.00 [13.60–87.60]) compared to culture-negative ones (n = 219; 2.83 [1.41–9.51], p < 0.0001, Benjamini-Hochberg correction method; Fig. 2E). The AUC (95% CI) was 0.85 (0.82–0.89) with a sensitivity (95% CI) of 0.96 (0.94–0.99) and a specificity (95% CI) of 0.47 (0.40–0.53), indicating a strong performance of the nasal IFN-I score in discriminating replicative viral infections, regardless of the virus involved. Using the 2.47 threshold, we obtained a PPV (95% CI) of 0.68 (0.63–0.73) and a NPV (95% CI) of 0.91 (0.86–0.96) to diagnose replicative viral infection. In addition, we found that the combination of nasal IFN-I score and viral load do not improve the performance of the nasal IFN-I score to predict viral replication (Supplementary Figure S1D).

Lastly, given recent studies highlighting the potential of IP-10 as a robust nasopharyngeal marker for RVI,^18^^,^^28^ we aimed to evaluate its performance compared to the nasal IFN-I score. IP-10 concentrations in NPS demonstrated a huge capacity to identify patients with RVI; with an AUC (95% CI) of 0.91 (0.88–0.93). However, assessing the ability of nasal IP-10 to discriminate between samples with viral culture-negative and positive, we found that the AUC (95% CI) was lower, at 0.72 (0.68–0.77), compared to the nasal IFN-I score of 0.85 (0.82–0.89) across all viruses tested, resulting in a AUC mean difference (95% CI) of 0.12 (0.077–0.17, p < 0.0001, DeLong test; Supplementary Figure S2). Furthermore, the performance of nasal IP-10 did not improve when AUCs were calculated for each virus individually (AUC <0.76) with a higher AUC mean difference compared to nasal IFN-I score of 0.21 (0.042–0.38) for IBV (Supplementary Figure S2). Taken together, using the samples classified as positive by the first-line tests, we report good performance of the nasal IFN-I score for assessing ongoing RVI.

### Improvement of viral detection of samples classified as negative by the first-line tests by the nasal IFN-I score

**Fig. 3:**
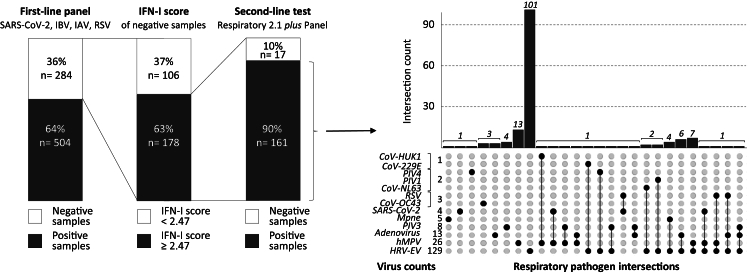
**Improvement of viral diagnosis using the nasal IFN-I score on samples initially classified as negative by first-line tests**. With the first-line test panel (SARS-CoV-2, IBV, IAV and RSV) 284 (36%) samples were classified as negative. Of these, 178/284 (63%) samples exhibited a nasal IFN-I score equal to or exceeding the fixed threshold of 2.47. A second-line test (BioFire**®** Respiratory Panel 2.1 *plus*) was carried out, one or more viruses were found in 161 (90%) samples with the second-line test, and 17 (10%) samples remained with negative results. The UpSetplot highlights the type and proportion of viruses found with the second-line test for the 161 positive samples (dot indicates mono-infection, several dots indicate co-infection). CoV, coronavirus. hMPV, human metapneumovirus. HRV-EV, human rhinovirus-enterovirus. IAV, influenza A virus. IBV, influenza B virus. IFN-I, type I interferon. Mpne, *Mycoplasma pneumoniae*. PIV, parainfluenza virus. RSV, respiratory syncytial virus. SARS-CoV-2, severe acute respiratory syndrome coronavirus 2.

In a subsequent step, we investigated how the nasal IFN-I score could enhance the diagnosis of RVIs in samples initially classified as negative for one of the four viruses targeted in the initial screening. Of the 788 samples collected, 284 (36%) were negative after first-line PCR tests, including 178 samples (63%) with a nasal IFN-I score greater than or equal to the 2.47 cut-off (Fig. 3).

Following the second-line test using the BioFire**®** Respiratory Panel 2.1 *plus*, 90% (161/178) of the samples initially classified as negative were found to contain at least one respiratory virus. Viral mono-infections were identified in 127 NPS (79%) with the most prevalent viruses being HRV/Enterovirus (EV) (80%, 101/127), hMPV (10%, 13/127), Parainfluenza 3 (PIV3) (3%, 4/127), ADV (2%, 3/127) and CoV-OC43 (2%, 3/127). Other viruses had a lower prevalence; SARS-CoV-2 (1%, 1/127) and Parainfluenza 4 (PIV4) (1%, 1/127). There were 34 samples with co-infections, each containing at least two viruses. Notably, NL63 and Parainfluenza 1 (PIV1) were only found in samples with co-infection (Fig. 3).

Among the 17 samples with a nasal IFN-I score greater than or equal to 2.47 but without virus detected after first- and second-line tests, 15 were analysed using metagenomics (for two samples the volume was insufficient for metagenomics; Table 1). Of these, six samples were identified as carriers of a pathogenic virus. Among these six samples, one contained human parvovirus B19, one contained human cytomegalovirus, and one contained human rhinovirus; and in three other samples there were reads belonging to the Picornaviridae family. Additionally, five other samples contained a virus from the Anelloviridae family, or human papillomavirus, although no respiratory pathogenic role was identified for these viruses. Ultimately, out of the nine subjects whose initial swabs did not reveal any pathogens, four exhibited nasal IFN-I score close to the cut-off of 2.47. The clinical trajectories of these nine patients involved intensive chemotherapy and haematological disorders (Table 1).

**Table 1:** Clinical presentation and pathogens detected by viral metagenomic on NPS from patients for whom first- and second-line tests were negative and with a nasal IFN-I score greater than or equal to 2.47.

Sample	Age, years	Hospital ward	Clinical history	Pathogen number of reads	Nasal IFN-I scores
A	63	Onco-haematology department	Acute myeloid leukaemia, bone marrow allograft the day before NPS collection, cystitis	No identification	3.45
B	00	ICU	Bronchiolitis with apnoea and desaturation	Human rhinovirus 6460 reads	16.05
C	09	Post-emergency unit	Febrile headache, abdominal pain, vomiting, fever	Picornaviridae 13 reads	5.79
D	12	Paediatric oncology unit	Rhabdomyosarcoma	Picornaviridae 176 reads	4.44
E	67	Onco-haematology department	Relapse of diffuse large-cell B lymphoma, recent initiation of immunosuppressants and chemotherapy	No identification	2.62
F	17	Onco-haematology department	Acute lymphocytic leukaemia (T lineage)	No identification	78.21
G	11	Paediatric oncology unit	Acute promyelocytic myeloid leukaemia	No identification	2.72
H	02	ICU	Hydrocephalus with external ventricular shunt infection leading to *Staphylococcus epidermidis* meningitis	No identification	2.53
I	10	Emergency room	Cyclic vomiting, high CRP at entry	Human parvovirus B19 30354905 reads	13.82
J	48	Onco-haematology department	Myelodysplastic syndrome, neutropenia, fever and desaturation	Picornaviridae 2457 reads	3.74
K	21	ICU	Coma due to multisubstance intoxication and hypothermia	No identification	3.59
L	71	Onco-haematology department	Diffuse large-cell B lymphoma, fever with digestive tract involvement	No identification	2.83
M	47	Onco-haematology department	Diffuse large-cell B lymphoma, fever, recent leukapheresis	No identification	2.76
N	67	Onco-haematology department	T-cell lymphoma relapse, initiation of chemotherapy	No identification	3.68
O	06	Post-emergency unit	*Staphylococcus aureus* osteomyelitis with septicaemia	Cytomegalovirus 16029 reads	79.01

In addition, we performed the second-line PCR testing on all negative samples with the first-line tests (n = 106, Fig. 3). We detected some viruses for 37 samples out of 106 first-line tests negative samples with nasal IFN-I score <2.47, mainly positive for HRV (28 out of 37). Nevertheless, we noticed that replicative viruses were only found in 3 out of 106 samples (Supplementary Figure S3).

## Discussion

In the present study, the nasal IFN-I score was found to be a relevant marker for detecting RVIs and enhancing the diagnosis of samples that initially tested negative using first-line tests (including SARS-CoV-2, IAV, IBV and RSV). Leveraging a substantial collection of NPS from a cohort of symptomatic patients with confirmed RVI after initial testing, we observed that the nasal IFN-I score was significantly higher in cases of viral infection compared to HCs. These findings corroborate our previous data, which demonstrated that patients with ongoing COVID-19 exhibited a markedly elevated nasal IFN-I score compared to a control group of HCs.^20^^,^^21^ Overall, this underscores the substantial potential of the nasal IFN-I score as a valuable universal marker for RVI.

The results confirmed that all respiratory viruses have the capacity to induce Type I IFN response, despite variations in the nasal IFN-I score according to the virus. However, it is worth noting that the lowest median nasal IFN-I score value was found for the SARS-CoV-2-positive NPS; this aligns with several studies indicating that the SARS-CoV-2 virus is a less potent inducer of the IFN-I response than the other respiratory viruses, such as RSV. This difference is attributed to the presence of proteins that alter both IFN-I production and signalling.^29^^,^^30^ In addition, we observed a very low nasal IFN-I score in some NPS detected positive for respiratory viruses using first-line panels, which can be explained by the presence of trace of nucleic acids from inactive virions,^6^ corresponding to low viral load. Host-based response tests could capture additional information about replicative viral status by relying on immune response features induced by viral infection. These results are in line with performance reported with others promising tools using blood samples such as FebriDx or MeMed.^31^^,^^32^ To go further, we analysed the nasal IFN-I score in relation to viral culture status. Viral culture is the gold standard method to determine the replicative capacity of a virus. We observed that the nasal IFN-I score was found to be able to distinguish (PCR-positive) non-replicative samples from replicative samples for SARS-CoV-2, IAV, IBV, and RSV. Most samples that tested positive initially but exhibited a low nasal IFN-I score (less than 2.47), yielded negative results in viral culture, indicating that the infection is no longer replicative. However, six of these samples showed positive viral cultures despite the low nasal IFN-I score. This discrepancy could be explained by the presence of auto-antibodies neutralising IFN-α and ω, thereby compromising early nasal IFN-I immunity during viral infection, by a defective immune response, or genetic disorders.^33^^,^^34^ However, it is important to note that viral culture may lack of sensitivity for samples with low viral load and it may fail due to sample collection, non-optimal cell lines and culture conditions and/or prolonged, particularly with labile viruses such as RSV.^35^, ^36^, ^37^, ^38^ Moreover, this technique is also time consuming as cells are incubated for days before the results are available. Due to these limitations, viral culture could not be considered as a practical approach to identify contagious subjects in a routine laboratory.

Furthermore, we compared the performance of the nasal IFN-I score with that of nasal IP-10, which had previously been identified as a promising diagnostic tool for RVI.^18^ However, its value in distinguishing replicative infections remained unexplored. To address this gap, we conducted a comparative analysis using the nasal IFN-I score. While both markers demonstrated similar capabilities in detecting RVI, the nasal IFN-I score outperformed nasal IP-10 in discerning replicative infections.^18^ This discrepancy could be explained by the inclusion of four ISGs in the nasal IFN-I score, allowing for a more comprehensive detection of the interferon response induced by viral infections, as opposed to the singular target represented by IP-10.

Interestingly, we also demonstrated that assessing the nasal IFN-I score can enhance the diagnosis of RVIs, particularly in cases where first-line diagnostic tests yield negative results (more than a third of the sample collection herein). In over half of these cases, the nasal IFN-I score was positive, indicating an active viral infection. This was corroborated by a second-line diagnostic test using a syndromic panel, which detected the presence of a virus in approximately 90% of these cases. Notably, HRV-EV mono-infection was the predominant finding, accounting for 70% of positive cases. Furthermore, viral metagenomic analysis revealed additional viruses in six patients, including one infection caused by parvovirus B19 and another by human cytomegalovirus.

The limited number of viruses targeted by the first-line tests recommended by health authorities leads to a high proportion of non-detected infections. Therefore, we propose adding the nasal IFN-I score as a screening tool to identify which samples should undergo broad viral PCR testing. This efficient strategy would enable to improve the detection of RVIs without diagnosis, optimising resource allocation by reducing the number of tests performed and mitigating associated costs. Additionally, metagenomic sequencing allowed us to refer samples that initially tested negative using both first- and second-line tests but exhibited a positive nasal IFN-I score for further exhaustive testing. Ultimately, this approach paves the way for enhancing preparedness in detecting emerging viruses in alignment with WHO priorities.^39^

The present study has some limitations. Firstly, the performance of the nasal IFN-I score was evaluated in a single hospital and needs to be validated in a multicentre study. The sample size for such a study should be determined based on the performance data reported here, and should cover multiple epidemic periods and include other countries, particularly those in the Southern Hemisphere, which have different virus circulation patterns.^40^ Conducting such a comprehensive study would allow us to better understand the marker's efficacy across diverse contexts and populations, as well as in non-hospital setting such as community laboratory. Secondly, nasal swab sampling occurred only once during hospital stays, without information on the time elapsed since symptom onset. Considering the rapid kinetics of the innate immune response, as documented in previous studies,^41^ understanding this dynamic is crucial to optimise the interpretation of host-based marker. A dedicated study with larger cohorts, ideally with repeated sampling and symptom monitoring, associated with an economic evaluation are essential to gain insight into the value of the test. In addition, the use of a composite gold-standard based on PCR, viral culture, viral metagenomics, and negative bacterial cultures could overcome the actual limits associated with tools involved in the RVI diagnosis.

In summary, the data emphasise the importance of incorporating the nasal IFN-I score into the clinical workflow alongside first-line tests recommended by guidelines. As a host response-based biomarker, the nasal IFN-I score provides a simpler alternative to current routine tests, such as culture or viral load assessment. Assessing the nasal IFN-I score holds significant promise for enhancing the detection of viral infections, crucial for improving patient care. Furthermore, this score can seamlessly be implemented into clinics and laboratories, as it is directly assessed from nasopharyngeal samples, the very site of active viral replication, already used for viral screening.

## Contributors

Conceptualisation, MMT, AG, KBP, FM, AF, STA.

Data curation, CG, DP, VC.

Formal analysis, MMT, DP, CG, AG, VC, GO, CD, QS, LG, SP, AGu, WM.

Funding acquisition, STA, AF, KBP, AB, BL, FM.

Investigation, STA, AF, KBP, AB, BL, FM.

Project administration, CD, AF, STA, KBP.

Resources, FL, DP, AG, AF, STA, FM.

Supervision, AG, WM, FM, AF, STA.

Validation, CG, MMT, WM.

Visualisation, WM, MMT.

Writing—original draft, MMT, DP, CG, AG, VC, BL, GO, LG, SP, KBP, AGu, WM, FM, AF, STA.

And writing—review & editing, MMT, DP, CG, AG, VC, BL, GO, FL, CD, QS, AB, LG, SP, KBP, AGu, WM, FM, AF, STA.

All authors were involved in drafting the manuscript and read and approved the final version of the manuscript.

## Data sharing statement

Requests for data should be made to the corresponding author. The datasets generated and analysed during the current study are available from the corresponding author on reasonable request. Metagenomic data have been submitted to the SRA database (PRJNA1171628).

## Declaration of interests

CG, KBP, VC, GO, MM, AGu, SP, LG, and AF are employees of bioMérieux SA, an in vitro diagnostic company. bioMérieux kindly provided kits (BioFire**®** Respiratory Panel 2.1 *plus* and FILMARRAY® IFN-I pouch prototype) for the study to DP, AG, BL, QS, AB, WM, FM and STA.

This funder of the study played a role in study design, data collection, data analysis, data interpretation, writing of the report, and decision to submit the manuscript for publication.

## Appendix A Supplementary data

Supplementary Figure S1 **Comparison of the performance of the nasal IFN-I score and the nasal viral load to discriminate samples with positive and negative viral culture among virus positive samples with first-line test.** Forest plots showing (A) median (IQR) of the nasal viral load (log10 cp/10^6^ cells) regarding its capacity to discriminate between nasal IFN-I score above (n = 282, filled circle) and below (n = 104, empty circle) the 2.47 cut-point for virus positive samples with first-line test to SARS-CoV-2 (n = 197), IAV (n = 63), IBV (n= 16), and RSV (n= 110), (B) median (IQR) of the nasal viral load (log10 cp/10^6^ cells) regarding its capacity to discriminate between samples with positive viral culture (n = 267, filled circle) and samples with negative viral culture (n= 179, empty circle) for SARS-CoV-2 (n = 166), IAV (n = 131), IBV (n = 33), and RSV (n = 116), (C) median (IQR) of the nasal IFN-I score regarding its capacity to discriminate between samples with positive viral culture (n=267, filled circle) and samples with negative viral culture (n= 179, empty circle) for SARS-CoV-2 (n= 166), IAV (n= 131), IBV (n = 33), and RSV (n = 116), and (D) exact AUC (95%CI) for the nasal viral load (dark circle), the nasal IFN-I score (grey circle) and the logistic regression (combined model) of the nasal IFN-I score combined with viral load (white circle) regarding their capacity to discriminate between samples with positive (n = 267) and negative (n = 179) viral culture for all virus types, SARS-CoV-2 (n = 166), IAV (n = 131), IBV (n = 33), and RSV (n = 116). Error bars indicate the 95% CIs. AUCs were compared using the DeLong test and were considered statistically significant if < 0.05. Error bars indicate the IQR. Median nasal viral loads were compared using the Wilcoxon-Mann-Whitney test and were considered significant if p.value < 0.05. AUC = area under the curve. IAV = influenza A virus. IBV = influenza B virus. IQR = interquartile range. RSV = respiratory syncytial virus. SARS-CoV-2 = severe acute respiratory syndrome coronavirus 2.

Supplementary Figure S2 **Performances comparison of the nasal IFN-I score and IP-10 concentration as a marker of replicative RVI.** Forest plot showing exact AUC (95% CI) for the nasal IFN-I score (empty square) compared to the IP-10 concentration (filled square) regarding their capacity to discriminate between samples with positive (n = 258) and negative (n = 227) viral culture for all virus types, SARS-CoV-2 (n = 202), IAV (n = 134), IBV (n = 37), and RSV (n = 112). Error bars indicate the 95%CIs. AUCs were compared using the DeLong test and were considered statistically significant if < 0.05. The mean differences (MD) between the AUCs, along with their 95% CI, are reported. AUC = area under the curve. IAV = influenza A virus. IBV = influenza B virus. IP-10 = IFN-y-inducible protein 10. MD = mean difference. RSV = respiratory syncytial virus. SARS-CoV-2 = severe acute respiratory syndrome coronavirus 2.

Supplementary Figure S3 **Viral culture status of samples initially classified as negative by first-line tests and with a nasal IFN-I score below the fixed threshold of 2.47**. Among samples negative with the first-line test, 106/284 (37%) samples exhibited a nasal IFN-I score below the fixed threshold of 2.47. A second-line test (BioFire® Respiratory Panel 2.1 plus) was carried out, one or more viruses were found in 37/106 (35%) samples with the second-line test, and 69/106 (65%) samples remained with negative results. Among the 37 samples positive with second-line test, 3/37 (8%) were found positive in viral culture and 34/37 (92%) were found negative. The pie chart highlights the type and proportion of viruses found with the second-line test for the 37 positive samples. Of note, the aetiology of the samples with viral culture-positive was solely attributed to HRV/EV viruses. In samples with co-infections, HRV/EV was respectively associated with hMPV, PIV3/ADV, and SARS-CoV-2. CoV = coronavirus. hMPV = human metapneumovirus. HRV-EV = human rhinovirus-enterovirus. IFN-I= type I interferon. SARS-CoV-2 = severe acute respiratory syndrome coronavirus 2.
