# Supplementary material for: Advancing respiratory virus diagnostics: integrating the nasal IFN-I score for improved viral detection
Source: eBioMedicine. 2024 Nov 21;110:105450. doi: 10.1016/j.ebiom.2024.105450 (PMC11617986; doi:10.1016/j.ebiom.2024.105450)
Supplement: Supplementary Figure S1 — Comparison of the performance of the nasal IFN-I score and the nasal viral load to discriminate samples with positive and negative viral culture among virus positive samples with first-line test. Forest plots showing (A) median (IQR) of the nasal viral load (log10 cp/106 cells) regarding its capacity to discriminate between nasal IFN-I score above (n = 282, filled circle) and below (n = 104, empty circle) the 2.47 cut-point for virus positive samples with first-line test to SARS-CoV-2 (n = 197), IAV (n = 63), IBV (n= 16), and RSV (n= 110), (B) median (IQR) of the nasal viral load (log10 cp/106 cells) regarding its capacity to discriminate between samples with positive viral culture (n = 267, filled circle) and samples with negative viral culture (n= 179, empty circle) for SARS-CoV-2 (n = 166), IAV (n = 131), IBV (n = 33), and RSV (n = 116), (C) median (IQR) of the nasal IFN-I score regarding its capacity to discriminate between samples with positive viral culture (n=267, filled circle) and samples with negative viral culture (n= 179, empty circle) for SARS-CoV-2 (n= 166), IAV (n= 131), IBV (n = 33), and RSV (n = 116), and (D) exact AUC (95%CI) for the nasal viral load (dark circle), the nasal IFN-I score (grey circle) and the logistic regression (combined model) of the nasal IFN-I score combined with viral load (white circle) regarding their capacity to discriminate between samples with positive (n = 267) and negative (n = 179) viral culture for all virus types, SARS-CoV-2 (n = 166), IAV (n = 131), IBV (n = 33), and RSV (n = 116). Error bars indicate the 95% CIs. AUCs were compared using the DeLong test and were considered statistically significant if < 0.05. Error bars indicate the IQR. Median nasal viral loads were compared using the Wilcoxon-Mann-Whitney test and were considered significant if p.value < 0.05. AUC = area under the curve. IAV = influenza A virus. IBV = influenza B virus. IQR = interquartile range. RSV = respiratory syncytial virus. SARS-CoV-2 = sev [file mmc1.pptx]

## Slide 1
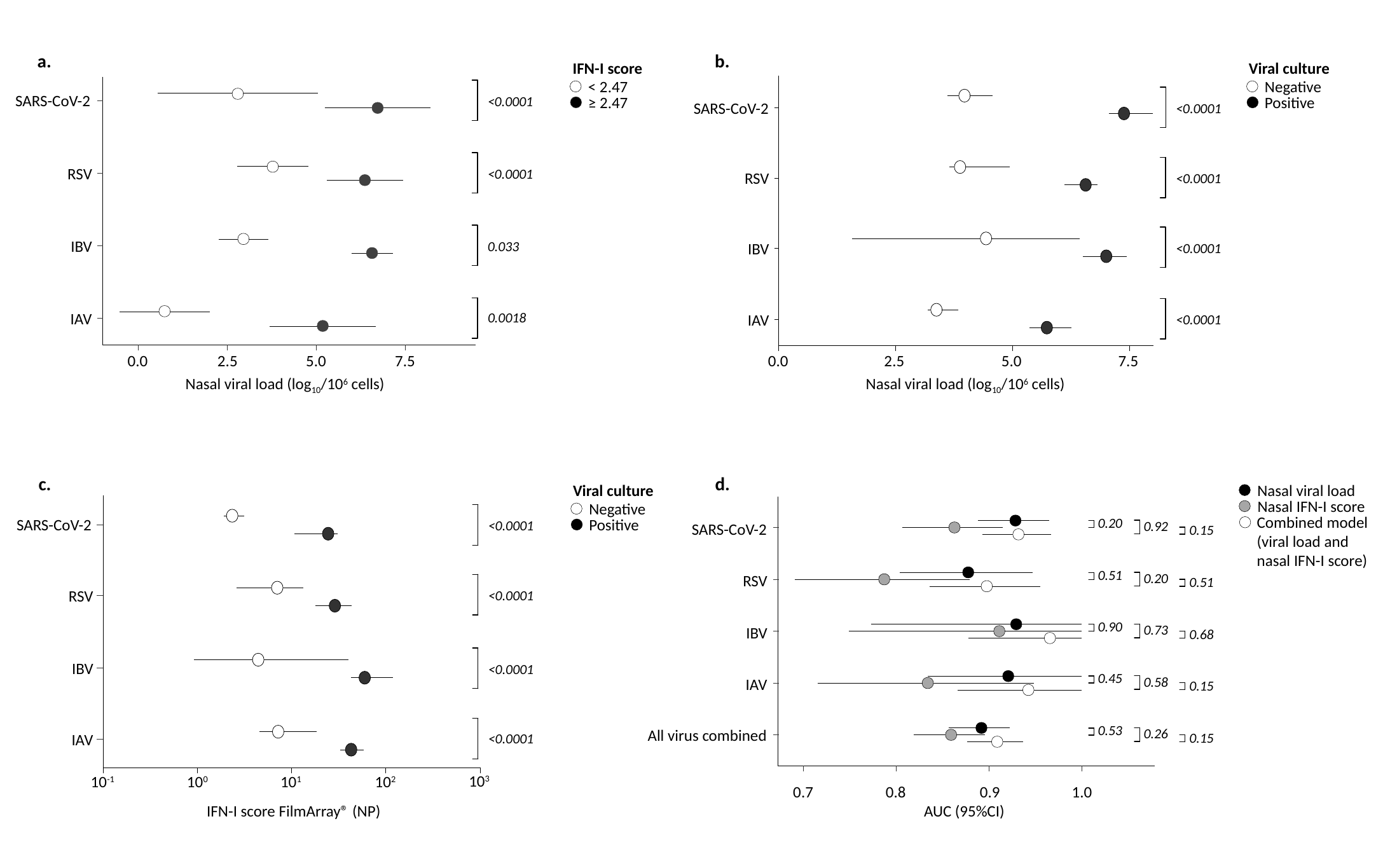

a.
b.
IFN-I score
< 2.47
≥ 2.47
Viral culture
Negative
Positive
<0.0001
SARS-CoV-2
<0.0001
SARS-CoV-2
<0.0001
RSV
<0.0001
RSV
0.033
IBV
<0.0001
IBV
0.0018
IAV
<0.0001
IAV
0.0
2.5
5.0
7.5
0.0
2.5
5.0
7.5
Nasal viral load (log10/106 cells)
Nasal viral load (log10/106 cells)
c.
d.
Viral culture
Negative
Positive
Nasal viral load
Nasal IFN-I score
Combined model
(viral load and nasal IFN-I score)
<0.0001
0.20
SARS-CoV-2
0.92
SARS-CoV-2
0.15
0.51
0.20
RSV
0.51
<0.0001
RSV
0.90
0.73
IBV
0.68
<0.0001
IBV
0.45
0.58
IAV
0.15
0.53
<0.0001
0.26
All virus combined
0.15
IAV
103
10-1
100
101
102
0.7
0.8
0.9
1.0
IFN-I score FilmArray® (NP)
AUC (95%CI)
