# Supplementary material for: Advancing respiratory virus diagnostics: integrating the nasal IFN-I score for improved viral detection
Source: eBioMedicine. 2024 Nov 21;110:105450. doi: 10.1016/j.ebiom.2024.105450 (PMC11617986; doi:10.1016/j.ebiom.2024.105450)
Supplement: Supplementary Figure S2 — Performances comparison of the nasal IFN-I score and IP-10 concentration as a marker of replicative RVI. Forest plot showing exact AUC (95% CI) for the nasal IFN-I score (empty square) compared to the IP-10 concentration (filled square) regarding their capacity to discriminate between samples with positive (n = 258) and negative (n = 227) viral culture for all virus types, SARS-CoV-2 (n = 202), IAV (n = 134), IBV (n = 37), and RSV (n = 112). Error bars indicate the 95%CIs. AUCs were compared using the DeLong test and were considered statistically significant if < 0.05. The mean differences (MD) between the AUCs, along with their 95% CI, are reported. AUC = area under the curve. IAV = influenza A virus. IBV = influenza B virus. IP-10 = IFN-y-inducible protein 10. MD = mean difference. RSV = respiratory syncytial virus. SARS-CoV-2 = severe acute respiratory syndrome coronavirus 2. [file mmc2.pptx]

## Slide 1
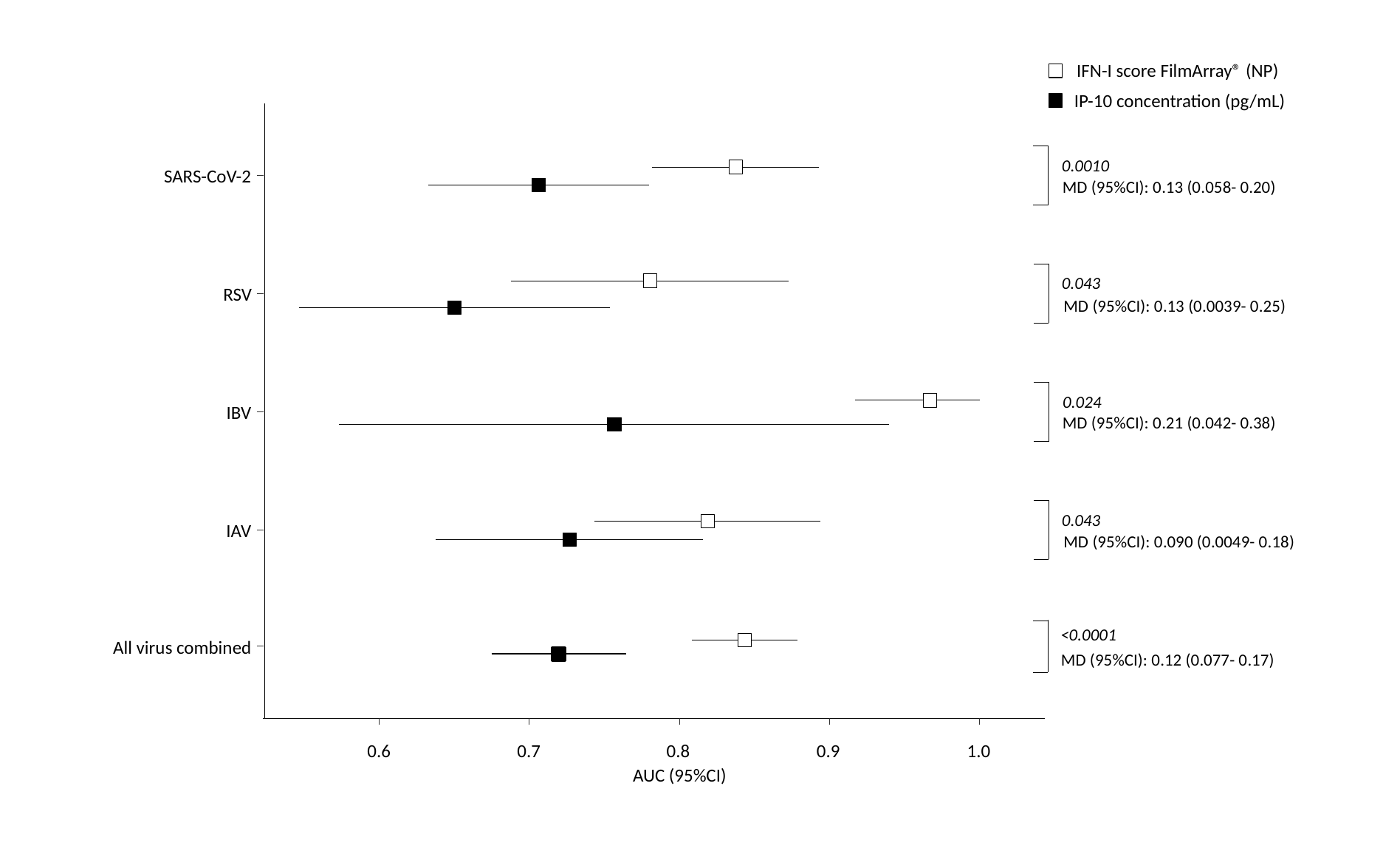

IFN-I score FilmArray® (NP)
IP-10 concentration (pg/mL)
0.0010
MD (95%CI): 0.13 (0.058- 0.20)
SARS-CoV-2
0.043
MD (95%CI): 0.13 (0.0039- 0.25)
RSV
0.024
MD (95%CI): 0.21 (0.042- 0.38)
IBV
0.043
MD (95%CI): 0.090 (0.0049- 0.18)
IAV
<0.0001
MD (95%CI): 0.12 (0.077- 0.17)
All virus combined
0.6
0.7
0.8
0.9
1.0
AUC (95%CI)
