# Supplementary material for: Advancing respiratory virus diagnostics: integrating the nasal IFN-I score for improved viral detection
Source: eBioMedicine. 2024 Nov 21;110:105450. doi: 10.1016/j.ebiom.2024.105450 (PMC11617986; doi:10.1016/j.ebiom.2024.105450)
Supplement: Supplementary Figure S3 — Viral culture status of samples initially classified as negative by first-line tests and with a nasal IFN-I score below the fixed threshold of 2.47. Among samples negative with the first-line test, 106/284 (37%) samples exhibited a nasal IFN-I score below the fixed threshold of 2.47. A second-line test (BioFire® Respiratory Panel 2.1 plus) was carried out, one or more viruses were found in 37/106 (35%) samples with the second-line test, and 69/106 (65%) samples remained with negative results. Among the 37 samples positive with second-line test, 3/37 (8%) were found positive in viral culture and 34/37 (92%) were found negative. The pie chart highlights the type and proportion of viruses found with the second-line test for the 37 positive samples. Of note, the aetiology of the samples with viral culture-positive was solely attributed to HRV/EV viruses. In samples with co-infections, HRV/EV was respectively associated with hMPV, PIV3/ADV, and SARS-CoV-2. CoV = coronavirus. hMPV = human metapneumovirus. HRV-EV = human rhinovirus-enterovirus. IFN-I= type I interferon. SARS-CoV-2 = severe acute respiratory syndrome coronavirus 2. [file mmc3.pptx]

## Slide 1
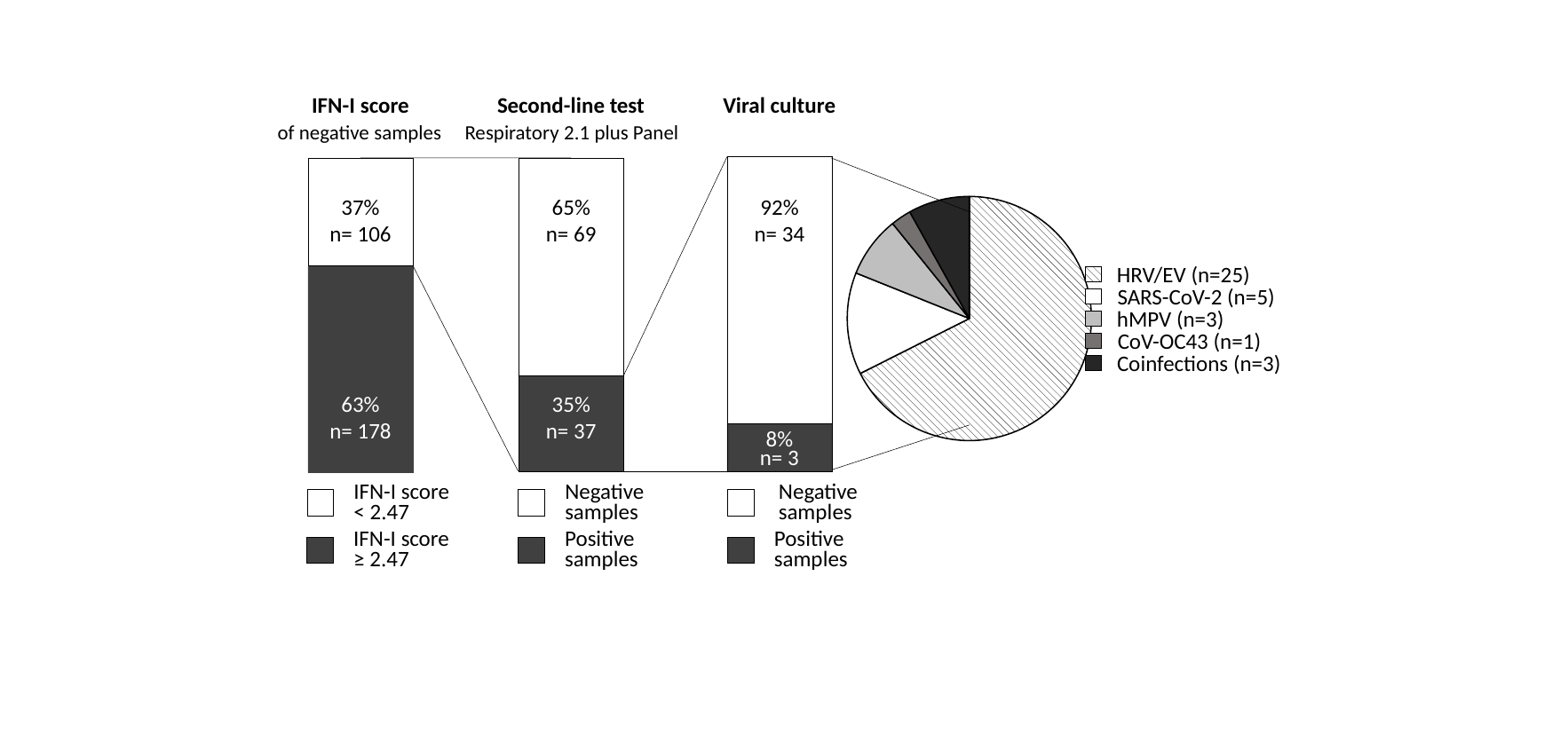

IFN-I score
Second-line test
Viral culture
of negative samples
Respiratory 2.1 plus Panel
92%
n= 34
37%
n= 106
65%
n= 69
### Chart
| Category | Ventes |
|---|---|
| HRV/EV (n = 25) | 25.0 |
| SARS-CoV-2 (n = 5) | 5.0 |
| hMPV (n = 3) | 3.0 |
| CoV-OC43 (n = 1) | 1.0 |
| Coinfections (n = 3) | 3.0 |HRV/EV (n=25)
SARS-CoV-2 (n=5)
hMPV (n=3)
CoV-OC43 (n=1)
Coinfections (n=3)
63%
n= 178
35%
n= 37
8%
n= 3
IFN-I score
< 2.47
Negative
samples
Negative
samples
IFN-I score
≥ 2.47
Positive
samples
Positive
samples
