# Supplementary material for: Advancing respiratory virus diagnostics: integrating the nasal IFN-I score for improved viral detection
Source: eBioMedicine. 2024 Nov 21;110:105450. doi: 10.1016/j.ebiom.2024.105450 (PMC11617986; doi:10.1016/j.ebiom.2024.105450)
Supplement: Supplementary Table S1 [file mmc4.docx]

**Supplementary table S1. Nasal IFN-I scores (medians [IQR]), AUCs (95%CI) obtained for each respiratory virus and multiple comparison of median nasal IFN-I score between each group.**

|  | **HCs** n= 53 | **SARS-CoV-2** n= 219 | **IAV** n= 143 | **IBV** n= 39 | **RSV** n= 126 |
| --- | --- | --- | --- | --- | --- |
| **AUC (95%CI)** | - | 0.87 (0.83-0.91) | 0.97 (0.95-0.99) | 0.95 (0.90-1.00) | 0.95 (0.91-0.98) |
| **Median [IQR]** | 1.09 [0.67-1.30] | 4.26 [1.74-24.60] | 27.50 [7.08-61.40] | 41.40 [11.10-81.40] | 17.40 [5.97-61.90] |
| **HCs *vs.*** | - | *<0.0001* | *<0.0001* | *<0.0001* | *<0.0001* |
| **SARS-CoV-2 *vs.*** |  | - | *<0.0001* | *<0.0001* | *<0.0001* |
| **IAV *vs.*** |  |  | - | *0.58* | *0.17* |
| **IBV *vs.*** |  |  |  | - | *0.17* |
| **RSV *vs.*** |  |  |  |  | - |

AUCs represent here the capacity of the nasal IFN-I score to discriminate HCs from patients with infection per virus. Multiples comparisons were performed using Kruskal-Wallis with Dunn’s test. Adjustment for multiple comparison was performed using Benjamini-Hochberg correction method and were considered statistically significant if p.adj <0.05 **(******, p.adj<0.0001).

AUC= area under the curve. HCs= healthy controls. IAV= influenza A virus. IBV= influenza B virus. RSV= respiratory syncytial virus. SARS-COV-2= severe acute respiratory syndrome coronavirus 2.
